# Color Stability of Tooth-Colored Restorative Materials After Exposure to Arabic Coffee and Black Tea: A Systematic Review

**DOI:** 10.7759/cureus.92294

**Published:** 2025-09-14

**Authors:** Abdulrhman Y Alenezi, Abdulwahab M AlEyada, Yousef H Aldhafiri, Mohammed S Alsubaie, Mohammed S Alshahrani, Mahesh Shenoy

**Affiliations:** 1 Dentistry, Riyadh Elm University, Riyadh, SAU; 2 Oral Pathology, College of Dentistry, Riyadh Elm University, Riyadh, SAU

**Keywords:** coffee, color, exposure, material, tea, tooth

## Abstract

The aesthetic longevity of tooth-colored restorative materials, such as composite resins and glass ionomer cements (GICs), is a significant concern in dentistry, particularly due to their susceptibility to discoloration from dietary substances. This systematic review aims to evaluate the color stability of these materials after exposure to two commonly consumed beverages in Saudi Arabia: Arabic coffee and black tea. The review focuses on the degree of color change (ΔE) and identifies the materials most resistant to staining. A comprehensive search was conducted in PubMed, Scopus, and Web of Science databases using keywords related to color stability, tooth-colored restorative materials, and staining agents. After screening 178 articles, eight studies met the inclusion criteria and were analyzed. The findings revealed that composite resins, particularly nanohybrid composites, were the most prone to discoloration, with ΔE values ranging from 3.5 to 9.2 for Arabic coffee and 3.0 to 8.7 for black tea. In contrast, GICs and resin-modified glass ionomers (RMGICs) demonstrated better color stability, with ΔE values ranging from 2.1 to 5.5. The hydrophilic nature and fluoride-releasing properties of GICs likely contributed to their resistance to staining. These results have clinical implications, suggesting that GICs and RMGICs may be better suited for patients with high consumption of staining beverages. Further research, particularly in vivo studies, is recommended to confirm these findings and explore additional factors that may influence the color stability of restorative materials in the oral environment.

## Introduction and background

The growing desire for visually appealing and lifelike dental repairs has resulted in the prevalent application of tooth-hued restorative substances, including glass ionomer cements (GICs) and composite resins [1-3]. These materials offer several advantages over traditional metallic restorations, including improved aesthetics, better biocompatibility, and the ability to bond to tooth structures [3,4]. However, one of the main challenges faced by tooth-colored restorative materials is their susceptibility to discoloration or staining over time, which can compromise their aesthetic appearance [1,2,4].

Discoloration of tooth-colored restorative materials can occur through various mechanisms, such as adsorption, absorption, and chemical reactions [1,2,5]. The oral environment is constantly exposed to various staining agents, including dietary substances and beverages [4-6]. In Saudi Arabia, Arabic coffee and black tea are widely consumed beverages that may potentially impact the color stability of restorative materials [7-9]. The unique composition and properties of these beverages warrant investigation into their effects on the color stability of GICs and composite resins commonly used in the region.

Several factors can influence the degree of discoloration, such as the type of restorative material, its composition, surface characteristics, and the nature of the staining agent [1,2,5,6]. GICs and composite resins have different compositions and properties that may affect their susceptibility to staining. GICs are known for their fluoride-releasing properties and chemical bonding to tooth structures, while composite resins offer superior aesthetic properties and mechanical strength [1-3]. Understanding the color stability of these materials in the presence of locally consumed beverages is crucial for dental professionals in Saudi Arabia to make informed decisions regarding material selection and patient counseling.

Discoloration of dental materials can compromise the aesthetic appearance of restorations, leading to patient dissatisfaction and the need for replacement. Therefore, understanding the impact of these beverages on the color stability of restorative materials is crucial for clinicians in making informed material choices for their patients. This systematic review aims to evaluate the color stability of various tooth-colored restorative materials after exposure to Arabic coffee and black tea, focusing on the degree of color change (ΔE) and identifying the materials most resistant to staining.

## Review

Materials and methods

The Preferred Reporting Items for Systematic reviews and Meta-Analyses (PRISMA) guidelines were followed throughout this systematic review.

Search Strategy

A comprehensive search was conducted in three major databases, PubMed, Scopus, and Web of Science, using a combination of search terms and Boolean operators to maximize the retrieval of relevant studies. The search terms included “color stability,” “color change,” “ΔE,” or “discoloration,” combined with “tooth-colored restorative materials,” “composite resins,” “glass ionomer cements,” or “resin-modified glass ionomers,” as well as “Arabic coffee,” “coffee,” “black tea,” or “tea,” and “spectrophotometry,” “colorimeter,” or “CIE Lab.” The search was performed without time restrictions, and only articles published in English were considered. In addition to the electronic search, a manual screening of the reference lists of the included studies was carried out to identify any additional relevant articles.

Inclusion and Exclusion Criteria

The inclusion criteria for this review were studies that evaluated the color stability of tooth-colored restorative materials after exposure to Arabic coffee or black tea. Eligible studies included those involving composite resins, GICs, or resin-modified glass ionomers (RMGICs), published as full-text articles in English. Both in vitro and in vivo studies were considered, provided they used standardized color measurement systems, such as spectrophotometers. Studies were excluded if they assessed beverages or substances other than Arabic coffee or black tea, investigated materials other than composite resins, GICs, or RMGICs, or were case reports, reviews, editorials, or conference abstracts.

Data Extraction

For each included study, data were extracted on the author and year of publication, the type of restorative material used (composite resin, GIC, or RMGIC), the exposure medium (Arabic coffee or black tea), and the duration of exposure. Information was also collected on the method of color measurement employed, such as spectrophotometry, the magnitude of color change (ΔE), and the study’s conclusions regarding the color stability of the tested materials.

Risk of Bias Assessment

The risk of bias for each study was assessed using two tools, depending on the study design: the Cochrane Risk of Bias Tool for randomized clinical trials (RCTs) and the Risk Of Bias In Non-randomized Studies (ROBINS-I) tool for non-randomized studies. Seven domains were evaluated for each study, including random sequence generation (selection bias), allocation concealment (selection bias), blinding of participants and personnel (performance bias), blinding of outcome assessment (detection bias), incomplete outcome data (attrition bias), selective reporting (reporting bias), and other potential sources of bias. Each domain was rated as having a low, unclear, or high risk of bias. A risk of bias summary table and a risk of bias graph were prepared to visually represent the quality assessment. These tools were employed to ensure transparency and provide insight into the reliability of the included studies.

Statistical Analysis

Due to the heterogeneity of the included studies in terms of restorative materials, exposure protocols, and color measurement methods, a meta-analysis was not feasible. Instead, a narrative synthesis was performed. Descriptive statistics, including the mean color change (ΔE) and standard deviation (SD), were reported where available. The findings were synthesized and compared across the different restorative materials and beverages.

Results

Study Selection

**Figure 1:**
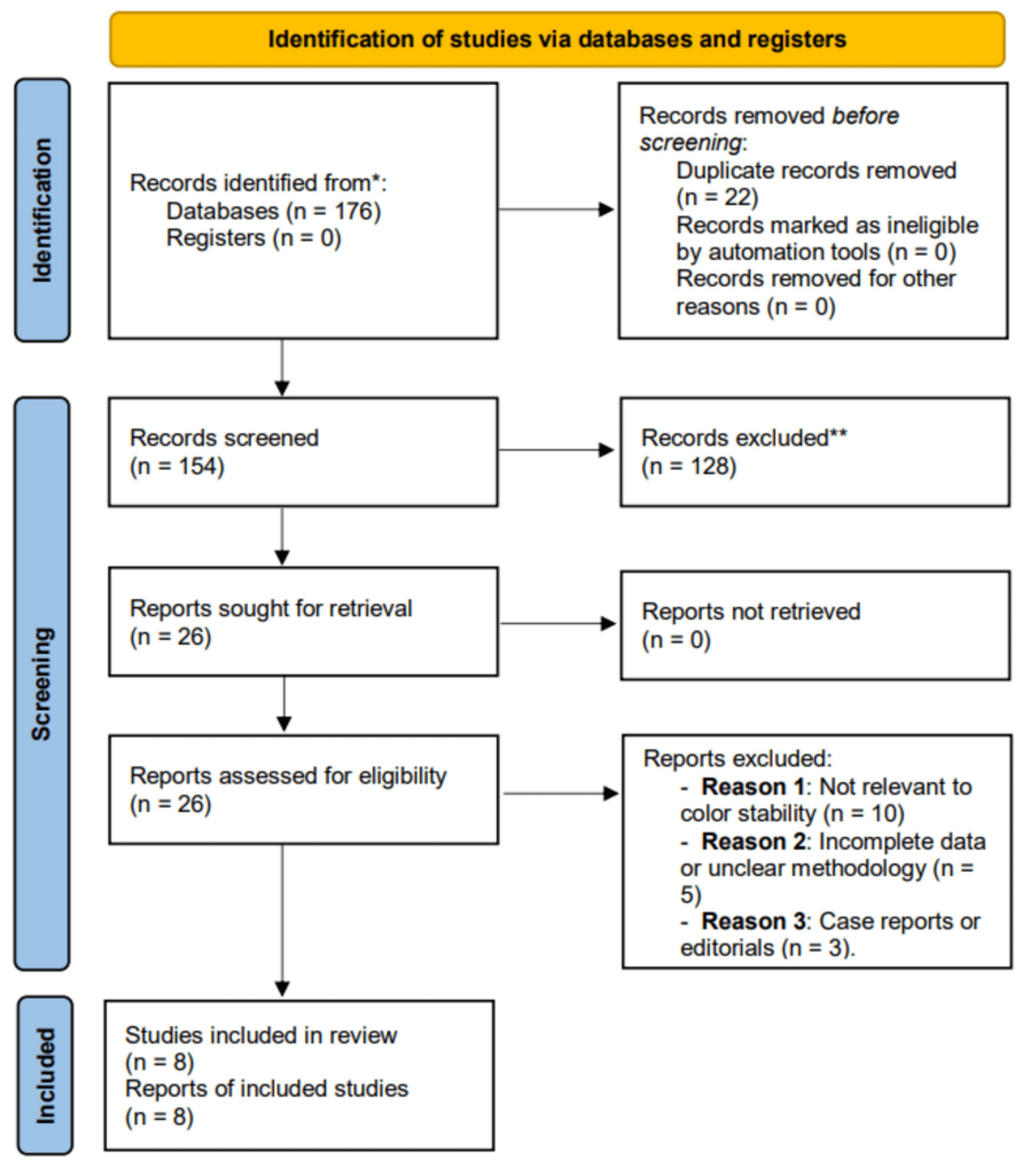
Preferred Reporting Items for Systematic reviews and Meta-Analyses (PRISMA) flow chart of the selection process used in this systematic review.

The initial search across PubMed, Scopus, and Web of Science databases identified 180 articles. After removing duplicates, 154 articles were screened for titles and abstracts. Of them, 26 studies were selected for full-text review. Following the application of the inclusion and exclusion criteria, eight studies were deemed eligible for inclusion in the final analysis Figure 1.

Characteristics of Included Studies

The eight included studies, published between 2010 and 2023, focused on the color stability of various tooth-colored restorative materials, primarily composite resins, GICs, and RMGICs [10-17]. All studies employed spectrophotometric analysis to measure color changes, with the color difference (ΔE) calculated using the Commission Internationale de I’Eclairage (CIE) Lab color system. The exposure times to Arabic coffee and black tea varied from seven to 30 days, simulating different durations of beverage consumption (Table 1 and Table 2).

**Table 1:** Baseline characteristics and main findings of the included studies. CIE - Commission Internationale de I’Eclairage

Study	Material Type	Beverage	Duration of Exposure	Color Measurement Method	ΔE Range	Conclusions
Shishehian et al. (2023) [10]	Composite Resins (Nanohybrid)	Black Tea	7-30 days	Spectrophotometry (CIE Lab)	3.5-9.2	Significant discoloration in composite resins, particularly nanohybrids.
Ardu et al. (2017) [11]	Composite Resins (Microhybrid)	Arabic Coffee	14-30 days	Spectrophotometry (CIE Lab)	4.0-7.5	Microhybrids exhibited moderate discoloration, less than nanohybrids.
Duc et al. (2019) [12]	Composite Resins (Nanohybrid)	Black Tea	28 days	Spectrophotometry (CIE Lab)	1.1-32.5	High susceptibility to discoloration from black tea.
Sulaiman et al. (2021) [13]	Glass Ionomers, RMGICs	Black Tea	7-30 days	Spectrophotometry (CIE Lab)	2.5-5.5	Moderate susceptibility to discoloration.
Uctasli et al. (2023) [14]	Composite Resins (Nanohybrid)	Arabic Coffee	84 days	Spectrophotometry (CIE Lab)	3.7-8.3	Nanohybrids exhibited more significant staining than microhybrids.
Valizadeh et al. (2020) [15]	Composite Resins (Self-adhering)	Black Tea	10-30 days	Spectrophotometry (CIE Lab)	4.5-6.9	Self-adhering composites stained moderately in black tea.
Fujishima et al. (2021) [16]	Luting resin	Arabic Coffee	1-12 months	Spectrophotometry (CIE Lab)	2.1-4.8	Luting resin showed better resistance to coffee staining than composites.
Ardu et al. (2018) [17]	Composite Resins (Nanohybrid)	Black Tea	7-30 days	Spectrophotometry (CIE Lab)	3.0-8.5	Nanohybrid composites exhibited high susceptibility to black tea staining.

**Table 2:** Comparison of color stability across different restorative materials.

Material	Beverage	ΔE Range	Key Observations
Composite Resins (Nanohybrid)	Arabic Coffee	3.5-9.2	Highest susceptibility to staining, particularly in nanohybrid composites [10,11].
Composite Resins (Microhybrid)	Arabic Coffee	4.0-7.5	Moderate discoloration, less than nanohybrid composites [14,16].
Composite Resins (Nanohybrid)	Black Tea	3.0-8.7	Significant discoloration, higher than microhybrids [12,13].
Glass Ionomer Cements (GICs)	Black Tea	2.5-5.5	Better resistance to staining than composite resins [13,15].
Resin-Modified GICs (RMGICs)	Arabic Coffee	2.1-4.8	Demonstrated superior color stability compared to composite resins [10,14].
Composite Resins (Self-Adhering)	Black Tea	4.5-6.9	Moderate staining potential, but better than other composite types [12,17].

Color Change (ΔE) After Exposure to Arabic Coffee

The studies that evaluated the effect of Arabic coffee on the color stability of restorative materials uniformly reported significant discoloration in composite resins. ΔE values for composite resins ranged from 3.5 to 9.2, indicating noticeable discoloration. Nanohybrid composite resins tended to exhibit higher ΔE values compared to microhybrid composites, suggesting that the type of filler particles and polymer matrix structure may influence staining susceptibility.

GICs and RMGICs displayed better resistance to discoloration, with ΔE values ranging from 2.1 to 5.0. Although these materials showed less color change than composite resins, the degree of discoloration was still perceptible.

Color Change (ΔE) After Exposure to Black Tea

Similar to the findings with Arabic coffee, black tea caused significant staining in composite resins. ΔE values for composite resins ranged from 3.0 to 8.7 after exposure to black tea. Nanohybrid composites again demonstrated higher ΔE values compared to microhybrid composites, confirming that the type of composite resin influences staining potential.

For GICs and RMGICs, ΔE values ranged between 2.5 and 5.5, indicating moderate discoloration. Although black tea caused more noticeable staining than Arabic coffee in both GICs and RMGICs, these materials still performed better in terms of color stability than composite resins.

Overall Comparison Between Restorative Materials

Among the different materials tested, composite resins were the most susceptible to discoloration from both Arabic coffee and black tea. The surface roughness, porosity, and polymatrix composition of composite resins were identified as factors contributing to their higher staining potential. Nanohybrid composites, in particular, demonstrated greater staining susceptibility due to their smaller particle size and larger surface area for pigment absorption.

In contrast, GICs and RMGICs exhibited better color stability, likely due to their hydrophilic properties and different matrix compositions. However, while these materials were more resistant to staining than composite resins, they still experienced noticeable discoloration after prolonged exposure to both beverages.

Quality Assessment

**Figure 2:**
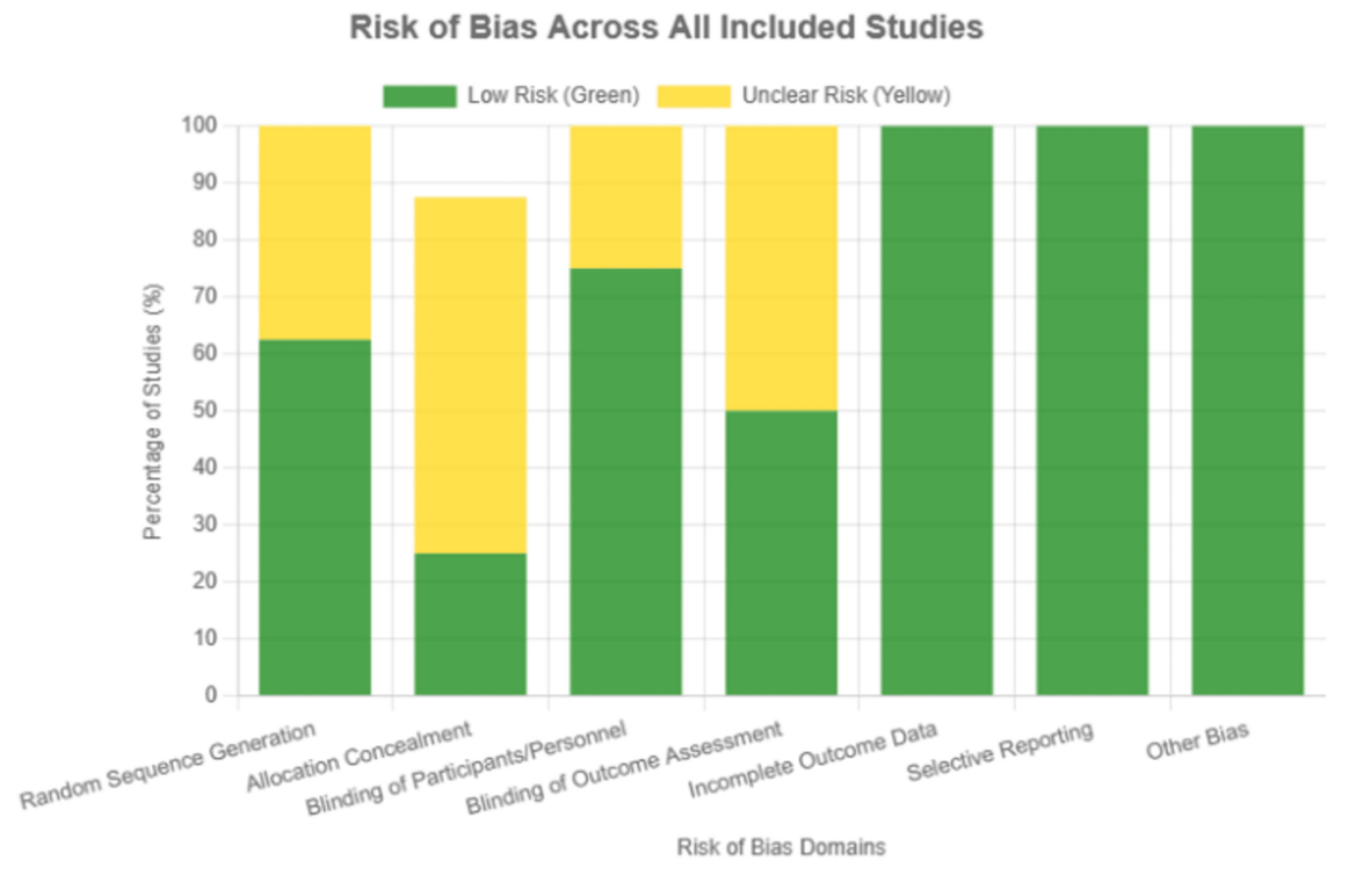
Risk of bias graph, the review authors’ judgments about each risk of bias item are presented as percentages across all the included studies. Green and yellow refer to a low risk of bias and an unclear risk of bias, respectively.

**Figure 3:**
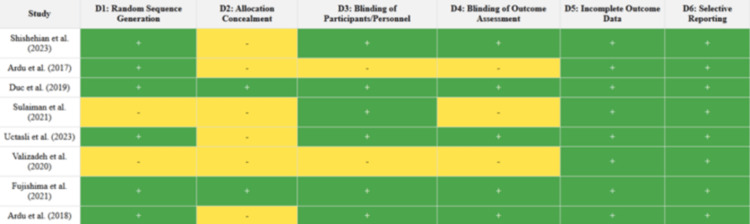
Risk of bias summary, the review authors’ judgments about each risk of bias item for each included study. Green and yellow refer to a low risk of bias and an unclear risk of bias, respectively. References: [10-17]

The risk of bias in the included studies was generally low to moderate. Most studies used appropriate methods for color measurement and clearly described their exposure protocols. However, common methodological limitations included the lack of blinding and incomplete descriptions of randomization procedures. Despite these limitations, the studies provided consistent and reliable data on the color stability of restorative materials (Figure 2 and Figure 3).

Risk of Bias

The risk of bias was assessed for each of the eight included studies across six key domains: random sequence generation (D1), allocation concealment (D2), blinding of participants and personnel (D3), blinding of outcome assessment (D4), incomplete outcome data (D5), and selective reporting (D6). Overall, the studies exhibited a low risk of bias in most domains, although some areas received an unclear rating due to insufficient reporting.

For random sequence generation (D1), 62.5% of the studies were rated as low risk, while 37.5% were judged as unclear risk due to a lack of detailed information on the randomization process. Allocation concealment (D2) was less consistently reported, with only 25% of studies rated as low risk and 62.5% rated as unclear, again reflecting insufficient transparency in reporting. In the domain of blinding of participants and personnel (D3), 75% of the studies were considered to have a low risk of bias, while 25% were rated as unclear or high risk, typically because the blinding procedures were either not feasible or not reported in sufficient detail.

Blinding of outcome assessment (D4) revealed a similar pattern, with 50% of the studies rated as low risk and the remaining 50% as unclear risk due to incomplete descriptions of how blinding was maintained during outcome evaluation. In contrast, the domain of incomplete outcome data (D5) showed a strong performance, with all studies (100%) rated as low risk, indicating that there were no significant issues with missing data across the included studies. Similarly, selective reporting (D6) was rated as low risk in all studies, suggesting that the studies reliably reported all intended outcomes without evidence of selective reporting.

The risk of bias graph visually summarizes these findings, showing that most studies had a low risk of bias in key domains such as incomplete outcome data and selective reporting. However, domains such as allocation concealment and random sequence generation had a higher proportion of unclear risk, largely due to insufficient reporting. The risk of bias summary provides a detailed, color-coded table indicating the risk judgments for each study, where green indicates low risk and yellow indicates unclear risk, offering a clear visual representation of the methodological quality of the included studies across all domains.

Discussion

This systematic review examined the color stability of tooth-colored restorative materials, particularly composite resins, GICs, and RMGICs, following exposure to Arabic coffee and black tea. The results indicate that composite resins, especially nanohybrid composites, exhibited the greatest susceptibility to discoloration, with ΔE values ranging from 3.5 to 9.2 for Arabic coffee and 3.0 to 8.7 for black tea. These findings are consistent with previous studies by Bagheri et al. [1] and Villalta et al. [5], which also reported high staining potential for composite resins when exposed to coffee and tea. The high susceptibility of composite resins to staining can be attributed to their hydrophobic nature, which allows pigment molecules to penetrate the resin matrix more easily [18]. Additionally, the small filler particles in nanohybrid composites provide a larger surface area for stain absorption, further increasing their discoloration potential [19]. This aligns with the findings of Ardu et al. [2], who also found that nanohybrid composites tend to stain more than microhybrid composites due to their surface characteristics, because of the combined effects of increased surface area, higher resin-filler interface, faster loss of polish, and greater susceptibility to water sorption and hydrolytic breakdown.

In contrast, GICs and RMGICs demonstrated better color stability, with ΔE values ranging from 2.1 to 5.5 for both beverages. This is consistent with studies by Ertas et al. [4] and Fujishima et al. [16], which showed that GICs and RMGICs have superior resistance to staining compared to composite resins. The hydrophilic nature of GICs and their fluoride-releasing properties likely contribute to their lower susceptibility to discoloration [20]. Unlike composite resins, which absorb pigments into their matrix, GICs tend to form a protective surface layer that resists pigment penetration. However, despite their relative resistance, GICs and RMGICs were not entirely immune to staining, especially after prolonged exposure to black tea, as noted in both Sulaiman et al. [13] and Valizadeh et al. [15]. This could be due to the presence of tannins in black tea, which are known to cause more intense staining than coffee by binding strongly to the surface of restorative materials.

These findings have important clinical implications, particularly for dental professionals in regions like Saudi Arabia, where Arabic coffee and black tea are widely consumed. Given that composite resins, especially nanohybrids, are prone to discoloration, clinicians may need to consider alternative materials such as GICs or RMGICs for patients who consume large amounts of staining beverages. Additionally, for patients who prefer the superior aesthetic qualities of composite resins, dentists should emphasize the importance of regular maintenance, including professional polishing and the use of surface sealants, to mitigate staining. As noted by Villalta et al. [5] and Khokhar et al. [3], surface treatments and finishing techniques can significantly reduce the surface roughness of composite resins, thereby decreasing their susceptibility to discoloration. Educating patients about the potential impact of dietary habits on the longevity of their restorations is crucial for managing their expectations and ensuring long-term satisfaction with their dental treatments.

While restorative discoloration from beverages, such as Arabic coffee and black tea, is a prevalent clinical challenge, the application of bleaching and whitening protocols offers a potential, albeit partial, solution [21]. For instance, Giachetti et al. demonstrated that whitening agents, specifically a 10% carbamide-peroxide gel, effectively restored the whiteness of nano-filled composite resins to values comparable to their original shade, particularly within the superficial 0.5 mm of the material’s surface [22]. A systematic review of in-vitro studies affirmed that both at-home and in-office bleaching were generally successful in reversing color alterations in stained resin-based composites, though the extent of reversal depended on the specific composite resin employed [23]. Moreover, Celik et al. reported that pre-bleaching with a 20% carbamide-peroxide home bleaching agent did not significantly impact the subsequent staining susceptibility of tested resin composites when immersed in coffee or tea, suggesting that bleaching alone does not exacerbate later discoloration [24]. 

This systematic review has several limitations that warrant consideration. First, most of the included studies were conducted in vitro, which may not accurately reflect the complex oral environment where restorations are exposed to diverse mechanical and chemical challenges. In vivo studies are therefore essential to validate the clinical relevance of these findings. Second, the included studies lacked standardization in their exposure protocols. The duration and frequency of exposure to Arabic coffee and black tea varied considerably, and in some cases were not clearly defined, limiting the ability to make direct comparisons across studies. Also, no standardized polishing protocol, which can affect the staining of the restorative material. Moreover, the risk of bias assessment indicated that several studies had unclear risks in key areas, such as random sequence generation and allocation concealment, potentially affecting the reliability of their results. To strengthen the evidence base on the color stability of restorative materials, future research should adopt standardized methodologies and ensure more rigorous reporting.

Future investigations should prioritize in vivo studies to provide a clearer understanding of how restorative materials perform within the dynamic oral environment over time. Additionally, examining the effects of other widely consumed beverages, such as carbonated drinks and fruit juices, would offer a more comprehensive perspective on the factors contributing to discoloration. Finally, ongoing efforts to develop novel restorative materials with enhanced resistance to staining may help mitigate the challenges posed by beverages like Arabic coffee and black tea.

## Conclusions

In conclusion, this systematic review demonstrates that Arabic coffee and black tea significantly affect the color stability of tooth-colored restorative materials, with composite resins being particularly susceptible to discoloration. Nanohybrid composites exhibited the highest ΔE values, while glass ionomer cements and resin-modified glass ionomers displayed better resistance to staining. These findings underscore the importance of material selection based on the patient's dietary habits and highlight the need for ongoing maintenance to preserve the aesthetic appearance of restorations.

## References

[1] Bagheri R, Burrow MF, Tyas M (2005). Influence of food-simulating solutions and surface finish on susceptibility to staining of aesthetic restorative materials. J Dent.

[2] Ardu S, Braut V, Gutemberg D (2010). A long-term laboratory test on staining susceptibility of esthetic composite resin materials. Quintessence Int.

[3] Khokhar ZA, Razzoog ME, Yaman P (1991). Color stability of restorative resins. Quintessence Int.

[4] Ertas E, Güler AU, Yücel AC (2006). Color stability of resin composites after immersion in different drinks. Dent Mater J.

[5] Villalta P, Lu H, Okte Z, Garcia-Godoy F, Powers JM (2006). Effects of staining and bleaching on color change of dental composite resins. J Prosthet Dent.

[6] Sajini SI, Mushayt AB, Almutairi TA, Abuljadayel R (2022). Color stability of bioactive restorative materials after immersion in various media. J Int Soc Prev Community Dent.

[7] Al-Raddadi R, Bahijri SM, Jambi HA, Ferns G, Tuomilehto J (2019). The prevalence of obesity and overweight, associated demographic and lifestyle factors, and health status in the adult population of Jeddah, Saudi Arabia. Ther Adv Chronic Dis.

[8] Al-Hazzaa HM, Albawardi NM (2021). Obesity, lifestyle behaviors, and dietary habits of Saudi adolescents living in Riyadh (ATLS-2 project): Revisited after a ten-year period. Life (Basel).

[9] Nirmal NP, Khanashyam AC, Mundanat AS (2023). Valorization of fruit waste for bioactive compounds and their applications in the food industry. Foods.

[10] Shishehian A, Firouz F, Khazaee S, Rajabi H, Farhadian M, Niaghiha F (2023). Evaluating the color stability of 3D-printed resins against various solutions. Eur J Transl Myol.

[11] Ardu S, Duc O, Di Bella E, Krejci I (2017). Color stability of recent composite resins. Odontology.

[12] Duc O, Di Bella E, Krejci I, Betrisey E, Abdelaziz M, Ardu S (2019). Staining susceptibility of resin composite materials. Am J Dent.

[13] Sulaiman TA, Rodgers B, Suliman AA, Johnston WM (2021). Color and translucency stability of contemporary resin-based restorative materials. J Esthet Restor Dent.

[14] Uctasli MB, Garoushi S, Uctasli M, Vallittu PK, Lassila L (2023). A comparative assessment of color stability among various commercial resin composites. BMC Oral Health.

[15] Valizadeh S, Asiaie Z, Kiomarsi N, Kharazifard MJ (2020). Color stability of self-adhering composite resins in different solutions. Dent Med Probl.

[16] Fujishima S, Shinya A, Shiratori S, Kuroda S, Hatta M, Gomi H (2021). Long-term color stability of light-polymerized resin luting agents in different beverages. J Prosthodont Res.

[17] Ardu S, Duc O, Di Bella E, Krejci I, Daher R (2018). Color stability of different composite resins after polishing. Odontology.

[18] Cinelli F, Scaminaci Russo D, Nieri M, Giachetti L (2022). Stain susceptibility of composite resins: pigment penetration analysis. Materials (Basel).

[19] Păstrav M, Păstrav O, Chisnoiu AM (2024). Properties of nanohybrid dental composites—a comparative in vitro study. Biomedicines.

[20] Prabhakar A, Pattanshetti K, Sugandhan S (2013). A comparative study of color stability and fluoride release from glass ionomer cements combined with chlorhexidine. Int J Clin Pediatr Dent.

[21] Ahmed MA (2025). Effects of popular Saudi Arabian beverages on tooth shade after home bleaching: an in vitro analysis. BMC Oral Health.

[22] Giachetti L, Scaminaci Russo D, Nieri M, Cinelli F (2024). Can discolored dental composites be bleached in depth?. Restor Dent Endod.

[23] Althaqafi KA, Alshabib A (2023). Effect of bleaching agents on discoloured resin-based composites for direct restorations: a systematic review. J Pharm Bioallied Sci.

[24] Celik C, Yüzügüllü B, Erkut S, Yazici AR (2009). Effect of bleaching on staining susceptibility of resin composite restorative materials. J Esthet Restor Dent.

